# Intraoperative identification and analysis of lymph nodes at laparoscopic colorectal cancer surgery using fluorescence imaging combined with rapid OSNA pathological assessment

**DOI:** 10.1007/s00464-017-5644-4

**Published:** 2017-06-22

**Authors:** Trevor M. Yeung, Lai Mun Wang, Richard Colling, Rebecca Kraus, Ronan Cahill, Roel Hompes, Neil J. Mortensen

**Affiliations:** 1 Nuffield Department of Surgical Sciences, University of Oxford, Oxford, OX3 9DU UK; 2 Department of Colorectal Surgery, Oxford University Hospitals NHS Foundation Trust, Oxford, UK; 3 Department of Cellular Pathology, Oxford University Hospitals NHS Foundation Trust, Oxford, UK; 4 Mater Misericordiae University Hospital and UCD School of Medicine & Medical Science, Oxford, UK

**Keywords:** Colorectal, Lymph Node, Fluorescence, OSNA, Imaging

## Abstract

**Background:**

Standard surgical practice for colorectal cancer involves resection of the primary lesion and all draining lymph nodes. Accurate intraoperative assessment of nodal status could allow stratified resectional extent. One-step nucleic acid (OSNA) can provide a rapid method of interrogating nodal tissue, whilst near-infrared (NIR) laparoscopy together with indocyanine green (ICG) can identify relevant nodal tissue intraoperatively.

**Methods:**

ICG was administered around the tumour endoscopically prior to the operation. Fluorescent nodes identified by NIR were marked and submitted for whole-node OSNA analysis. Further fresh lymph nodes dissected from the standard resection specimen were examined and analysed by both conventional histology and OSNA. In addition, the status of the fluorescent nodes was compared to that of non-ICG nodes to assess their predictive value.

**Results:**

Sixteen patients were recruited with a total final lymph node count of 287. 78 fresh lymph nodes were identified on fresh dissection for both histological and OSNA assessment with an analytical concordance rate of 98.7% (77/78). OSNA sensitivity was 1 (0.81–1, 95% CI) and specificity 0.98 (0.91–1, 95% CI). Six patients had a total of nine nodes identified intraoperatively by ICG fluorescence. Of these nine nodes, one was positive for metastasis on OSNA. OSNA analysis of the ICG-labelled node matched the final histological nodal stage in 3/6 patients (two being N0 and one N1). The final pathological nodal stage of the other three was N1 or N2, while the ICG nodes were negative.

**Conclusion:**

OSNA is highly concordant with standard histology, although only a minority of nodes identifiable by full pathological analysis were found for OSNA on fresh dissection. OSNA can be combined with NIR and ICG lymphatic mapping to provide intraoperative assessment of nodal tissue in patients with colorectal cancer.

Currently, the standard operation for colorectal cancer involves the excision of the primary lesion together with all its draining lymph nodes, although there is no treatment benefit from the removal of normal (i.e. tumour negative) lymph nodes. Colorectal cancer screening programmes mean many patients are diagnosed with true early stage (i.e. N0) disease. Recently, extended lymphadenectomy has been proposed for colonic cancers, while others are considering lateral iliac nodal clearance for selected patients with rectal cancer. A facility to confidently assign nodal status intraoperatively independent of the performance of the radical resection could allow stratification of operative extent by disease stage without compromising prognostic or therapeutic value.

One-step nucleic acid amplification (OSNA) is a rapid mRNA assay that can detect colorectal micrometastases in lymph nodes based on cytokeratin 19 (CK19) levels within 20 min of their removal [1–3]. Although its use to date has predominantly been from the perspective of entire node basin assessment, its technical capability provides results within a timeframe that could guide intraoperative decision making. OSNA requires fresh nodal tissue; therefore either the entire nodal basin needs removal for fresh tissue dissection or a selective, representative amount of nodal tissue may be identified by the surgeon. Our group and others have previously demonstrated that peritumoral submucosal injection of indocyanine green (ICG) with near-infrared (NIR) laparoscopy provides lymph node visualisation during colorectal cancer resections [4–6]. However, the pathological relevance of these fluorescent lymph nodes is currently not clear.

This pilot study assesses the concordance between standard histology and OSNA for the analysis of lymph nodes in combination with NIR lymphangiography as proof-of-principle that current technology can support focused intraoperative node interrogation. As large patient numbers are needed for definitive conclusions, this work aimed to clarify the necessary protocols and thresholds to inform a definitive trial.

## Methods

This was an open label, prospective trial assessing the feasibility of using OSNA analysis of fresh lymph nodes in combination with ICG and NIR fluorescence for the identification of lymph nodes during laparoscopic colorectal surgery. The primary objective was to compare of OSNA assessment of lymph nodes with standard histopathology to verify that this technology could be employed intraoperatively in lieu of standard pathological assessment. In addition, the predictive value by OSNA of the ICG-labelled nodes with respect to indicating the pathological nodal stage of the patient was examined to determine whether or not such identified nodes could be deemed sentinel or significant.

This study was reviewed and given a favourable opinion by the Outer North London Research Ethics Committee (REC Reference No: 10/H0724/13). All patients included in the study had agreed to participate following provision of fully informed consent. Inclusion criteria were any patients aged 18 or above, diagnosed with colorectal neoplasia requiring surgical excision by laparoscopic means. Exclusion criteria included any female participant who was pregnant, lactating or planning pregnancy during the course of the study, allergy to any of the compounds being used for lymphatic mapping including indocyanine green, and patients undergoing purely palliative surgery.

**Fig. 1:**
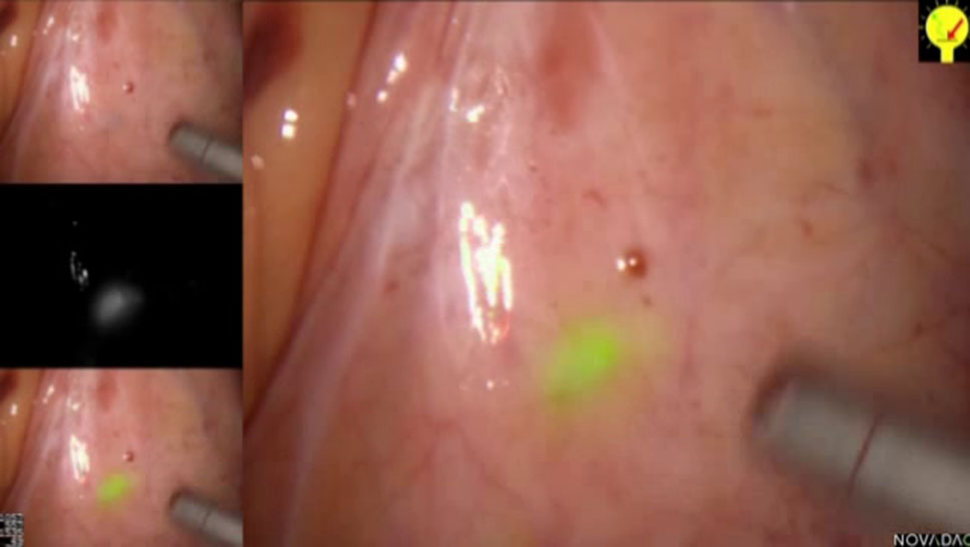
Laparoscopic visualisation of mesocolic tissue under white light (*top left*) and under fluorescence (*middle left*). White light and fluorescence images marged (*bottom left and right*)

For patients with left-sided colonic and rectal lesions, an enema was administered on the morning of surgery. Immediately after induction of anaesthesia and patient positioning and prior to any incision, a flexible endoscope was passed to point of the tumour to allow a 1–2 ml submucosal injection of ICG (2.5–5 mg/ml) in four points around the tumour. Laparoscopy was then performed, and the mesocolic and mesorectal tissue was examined by both direct visualisation and under near-infrared (NIR) illumination (PINPOINT, Novadaq) and any identifiable lymph nodes were marked in situ by clipping or excised by “berry picking” as previously described (Fig. 1). The fluorescence of the nodes was checked for up to 20 min after ICG injection. Time was limited due to the need to complete the operation and to proceed to other cases on the list the same day. All patients underwent standard colorectal cancer operation in the conventional manner.

The colorectal specimens were sent fresh for macroscopic examination by a consultant pathologist to confirm the lack of serosal and resection margins involvement before proceeding with fresh lymph node dissection. According to manufacturer’s specification, only lymph nodes that were greater than 10 mm or had a minimum weight of 0.05 g were regarded as suitable for OSNA analysis. Thus, lymph nodes greater than 10 mm found by fresh dissection were each labelled as “fresh lymph node”. Each fresh lymph node was bisected with half kept for OSNA and the other half by standard H&E microscopic evaluation (i.e. formalin-fixed, processed and a single H&E-stained 4-µm-thick tissue section mounted on glass slide). Those smaller lymph nodes dissected fresh that did not meet the criteria for OSNA were formalin-fixed and examined by standard H&E section. Individual ICG-identified lymph nodes underwent whole-node OSNA without H&E microscopic evaluation. Each colorectal specimen, after fresh nodal retrieval, was fixed in formalin and processed for standard histological examination.

For OSNA analysis, the whole ICG-identified nodes and half a node from each pathologist-dissected fresh lymph node were cleaned of fat and frozen in liquid nitrogen and stored at −80 °C for processing at a later time. Lymph nodes weighing between 0.05 g and 0.6 g were processed singularly. Larger nodal tissue was dissected for processing in smaller pieces. Smaller ‘underweight’ lymph nodes were combined for processing. Lymph nodes were placed in homogenised in lysis buffer (Lynorhag; Sysmex) and then centrifuged. Lysate was then extracted for CK19 mRNA RT-LAMP in the RD-100i system (Sysmex) using the Lynoamp (Sysmex) reagent kit, according to the manufacturer’s instructions’. A CK19 copy number ≥250/µL was considered positive based on the manufacturers recommendation. A calibration run and standard curve was set before each batch run of clinical cases. Any lymph node with negative histology but positive OSNA underwent multiple H&E-stained level sections for histology of the entire remaining formalin-fixed nodal tissue.

All subjects were followed at 2 weeks and 3 months postoperatively for any evidence of adverse reactions. These visits were scheduled to coincide with the patients routine postoperative follow-up.

## Results

16 patients were eligible and consented for the trial. All 16 patients completed all aspects of the study during the timeframe. Their average age was 64.5 years (range 43–78). All underwent laparoscopic resections for colorectal cancer, four having sigmoid colectomy, eleven having anterior resection and one having a panproctocolectomy. Tumour stage details are as follows: one patient with T1 (Sm3), five with T2, eight with T3 and two with T4. The total lymph node count (including ICG-identified nodes, freshly dissected nodes and nodes identified on histological sectioning) was 287 from 16 patients, ranging from 9 to 32 nodes per patient (median 16.5). OSNA analysis was compared with standard histological assessment in all patients. 78 fresh lymph nodes were identified by the pathologist in addition to nine nodes being identified in vivo by ICG-mapping. Histological and OSNA analysis were concordant for 77 out of 78 lymph nodes (98.7%) (Table 1). One lymph node positive by OSNA (1.3%) was found to be negative for metastases on standard histology. With further histological sectioning of this lymph node with extra levels, it was later confirmed to have metastatic involvement. Overall, OSNA had a sensitivity of 1 (0.81–1, 95% CI) and a specificity of 0.98 (0.91–1, 95% CI). Its positive predictive value was 0.94 (0.73–1, 95% CI) and negative predictive value was 1 (0.94–1, 95% CI).

**Table 1:** Comparing histology with OSNA analysis of retrieved fresh lymph nodes

	Histology +	Histology −	Total
OSNA +	16	1	17
OSNA −	0	61	61
Total	16	62	78

Out of the total sixteen patients in the study, a total of nine nodes were identified by ICG in six patients (37.5% detection rate, mean number of nodes per patient when identifiable 1.5, range 1–3). The other 10 patients did not have any fluorescent nodes seen intraoperatively. Of the nine ICG-labelled nodes, one was positive for metastasis on OSNA analysis. The OSNA analysis of the ICG-mapped node matched the final histological nodal stage in three cases (two being were N0 and one N1). For the other three patients, the OSNA analysis of the ICG node was not concordant with the basin final stage, in each of these the OSNA analysis was negative and the final pathology was either N1 or N2. There were no adverse side effects following the peritumoural injection of ICG in our cohort of patients.

## Discussion

In this pilot study, we have verified that OSNA is as accurate as standard H&E examination in lymph nodes submitted to both techniques. For patients with colorectal cancer, OSNA has been found to be highly sensitive (96.4%) and specific (100%) for the detection of lymphatic metastases [3], and the results of our study are consistent with this. However, we have found that only a minority of nodes ultimately available for scrutiny by standard pathological assessment were identifiable using ICG fluorescence, meaning this technique only provides a sampling.

We have demonstrated proof of principle that the use of fluorescence imaging to identify colorectal lymph nodes during surgery may be combined with OSNA to provide accurate interrogation of the oncological status of these specific lymph nodes. Such lymph nodes in this study could ,however, clearly not be considered sentinel or representative. With further refinement in technique and with more experience using this combined technology, OSNA could eventually be used intraoperatively with ICG-analysis for the identification of node-positive patients that would affect surgical decision making (i.e. either the extent of next level lymphadenectomy or utilise peritoneal chemotherapy perhaps). However, at this stage, from this data, neither should be used to predict node-negative patients in order to limit dissection extent.

The limitations of our study are that it involved a smaller number of patients then expected due to the considerable logistic and practical issues experienced. Given that the elective surgical work at our hospital is at a site separated from the pathological laboratories and given the vagaries of theatre list timing regarding specimen extraction, it proved difficult for the consultant pathologist to be available for the fresh resection tissue examination. Fluorescence node detection rates were also far below the levels we and others have previously found using the same technique and technology and likely reflects a difficulty in having similar access to endoscopic expertise across all cases. Nodal detection rates are likely to improve with greater experience and more cases. Furthermore, even if a minority of patients benefit from this technology, this could still have a significant impact on surgical decision making during their operation. We know from previous work that improving fluorescence node detection rates is the first step towards the elegant concept for OSNA identification of node negative patients during surgery. Doing so would allow determination of whether ICG identifiable lymph nodes are truly representative sentinel nodes as this is key information to any consideration of their use in limiting operative dissection using this technology.
